# Performance of QuantiFERON-TB Gold Plus for detection of latent tuberculosis infection in pregnant women living in a tuberculosis- and HIV-endemic setting

**DOI:** 10.1371/journal.pone.0193589

**Published:** 2018-04-04

**Authors:** John König Walles, Fregenet Tesfaye, Marianne Jansson, Taye Tolera Balcha, Niclas Winqvist, Mestawet Kefeni, Sileshi Garoma Abeya, Feleke Belachew, Erik Sturegård, Per Björkman

**Affiliations:** 1 Clinical Infection Medicine, Department of Translational Medicine, Lund University, Malmö, Sweden; 2 Department of Infectious Diseases, Central Hospital, Kristianstad, Sweden; 3 Medical Microbiology, Department of Laboratory Medicine, Lund University, Lund, Sweden; 4 Armauer Hansen Research Institute, Addis Ababa, Ethiopia; 5 Skåne Regional Office for Infectious Disease Control and Prevention, Malmö, Sweden; 6 Adama Regional Laboratory, Adama, Ethiopia; 7 Adama Hospital Medical College, Adama, Ethiopia; Universita degli Studi di Palermo, ITALY

## Abstract

We evaluated the performance of QuantiFERON-TB Gold Plus (QFT-Plus), which includes two *Mycobacterium tuberculosis* antigen formulations (TB1 and TB2), for detection of latent tuberculosis infection during pregnancy. Eight-hundred-twenty-nine Ethiopian pregnant women (5.9% HIV-positive) were tested with QFT-Plus, with bacteriological sputum analysis performed for women with clinically suspected tuberculosis and HIV-positive women irrespective of clinical presentation. QFT-Plus read-out was categorized according to the conventional cut-off (0.35 IU/ml) for both antigen formulations. In addition, we analysed the distribution of QFT-Plus results within a borderline zone (0.20–0.70 IU/ml), and interferon-γ response in relation to HIV infection and gestational age. Two-hundred-seventy-seven women (33%) were QFT-Plus-positive (HIV-positive 16/49 [33%]; HIV-negative 261/780 [33%]). There was a strong agreement between the two antigen formulations (κ = 0.92), with discordant results in 29 cases (3.5%). Whereas discordant QFT-Plus results were rare in pregnancy, several results with both TB1 and TB2 within the borderline range were observed (11/49 [22%] vs. 43/780 [5.5%] in HIV-positive and HIV-negative women, respectively; p<0.0001). HIV-positive women had lower absolute interferon-γ levels (TB1: 0.47 vs. 2.16 IU/ml; p<0.001, TB2: 0.49 vs. 2.24 IU/ml, p<0.001, considering results ≥0.20 IU/ml) compared to HIV-negative women. QFT-Plus-positive women who submitted samples at later stages of pregnancy had lower mitogen- (p<0.001) but higher TB-antigen-specific (p = 0.031 for TB1, p = 0.061 for TB2) interferon-γ response. Considering their lower capacity to produce TB-specific interferon-γ, a lower cut-off level for defining QFT-Plus-positivity may be considered in HIV-positive pregnant women.

## 1. Introduction

Most persons infected with *Mycobacterium tuberculosis* (Mtb) have latent tuberculosis infection (LTBI) in which viable bacteria are confined to granulomas regulated by host immunity [1]. Reactivation of LTBI can lead to active tuberculosis (TB), and is often related to various types of immunosuppression, especially HIV infection [2]. Pregnancy is a physiologic condition during which alterations of the immune system occur, and become more profound with increasing gestational age [3]. Moreover, many infections are more likely to have severe manifestations in the third trimester of gestation [4]. It has also been suggested that pregnancy may confer an increased risk of LTBI reactivation [5,6]. Furthermore, since active TB is associated with adverse pregnancy outcomes [7–9], TB screening is recommended during antenatal care.

For several reasons the diagnosis of LTBI is challenging. Firstly, no diagnostic gold standard exists for this condition. Secondly, all available methods rely on detection of cell-mediated immune response to Mtb, which explains why performance is reduced in immunosuppressed individuals, who are also at increased risk of progression to active disease. Finally, current tests cannot distinguish between true latent infection, i.e. persistence of contained viable bacteria, cleared TB infection and active disease.

Interferon-γ release assays (IGRAs) are increasingly used for LTBI diagnosis in high-income countries [10]. In the QuantiFERON-TB Gold In-Tube (QFT-GIT) assay the concentration of interferon-γ (IFN-γ) is measured in plasma after incubation with Mtb antigen peptides (early secretory antigenic target 6-kD protein [ESAT-6], culture filtrate protein 10 [CFP-10] and tuberculosis-7.7 [TB7.7]). The assay also encompasses a negative control (nil), and a positive control using phytohemagglutinin (mitogen). The performance of QFT-GIT depends on the stimulation of CD4+ T-cells, which limits its performance in HIV-positive subjects, who have reduced capacity for IFN-γ secretion from CD4+ T-cells [11]. A new version of the QuantiFERON assay, QuantiFERON-TB Gold Plus (QFT-Plus), includes two Mtb antigen tubes; TB1 (containing ESAT-6 and CFP-10 peptides), and TB2 (containing ESAT-6 and CFP-10 peptides, as well as shorter peptides of the same antigens designed to specifically stimulate CD8+ T-cells), and the TB7.7 antigen has been removed. The test is considered positive if IFN-γ ≥ 0.35 IU/ml in either TB1 or TB2. It has been suggested that this modification could improve sensitivity in immunosuppressed subjects, moreover, stimulation of CD8+ T-cells might improve sensitivity in recently infected individuals [12]. The sensitivity of tests for LTBI (including both the tuberculin skin test [TST] and QFT-GIT) may be reduced during pregnancy due to physiological immune modulation [13,14], in particular among HIV-positive pregnant women [15], in whom high rates of indeterminate QFT-GIT results have been reported [16]. Specific stimulation of CD8+ T-cells might be relevant during pregnancy due to the suppression of the TH1-response in pregnancy [17,18] and decrease in absolute CD4+ T-cell counts [19–21], while CD8+ T-cell counts remain unchanged [20].

Several studies performed in low TB-endemic settings have found considerable variability on repeated QFT-GIT testing in subjects with initial results around the recommended cut-off level of 0.35 IU/ml [22–24]. This phenomenon has led many authors to recommend re-testing of such borderline results, although different definitions of borderline ranges exist [22,24–29]. The practice of re-testing subjects with initial QFT-GIT results close to the cut-off was proposed by the Centre of Disease Control (CDC) in 2010, although no threshold levels for defining a borderline interval were specified [30]. For this study, we have chosen to explore a borderline range of 0.20–0.70 IU/ml, which recently was evaluated for QFT-GIT with respect to TST concordance and incidence of active TB [31].

Recently, it has been suggested that the optimal cut-off level for defining positive results might be lower than 0.35 IU/ml in HIV-positive subjects [32], due to impaired effector responses to Mtb antigens.

The QFT-Plus assay has not been assessed for detection of LTBI in pregnant women. Furthermore, to our knowledge, the distribution of QFT borderline results has not been explored among women tested during pregnancy.

We here present an evaluation of the performance of QFT-Plus in a cohort of pregnant women in Ethiopia. In this study, we have aimed to determine the additional diagnostic yield of TB2 antigen stimulation and the agreement between TB1 and TB2 results, with particular regard to HIV serostatus. Furthermore, we have analysed the distribution of QFT-Plus results within a borderline range, as well as absolute Mtb-stimulated IFN-γ levels in relation to HIV infection and gestational age.

## 2. Materials and methods

### 2.1 Study participants

This study was based on data collected between November 2015 and August 2016 from the first 829 consecutively recruited participants in an ongoing prospective cohort study on TB in women of fertile age conducted at three public health facilities in Adama, Ethiopia. Pregnant women attending any of the study sites for their first antenatal care visit for the current pregnancy were eligible for inclusion in the cohort study after giving written informed consent. At inclusion, data was collected by trained staff using standardized questionnaires covering socioeconomic and demographic characteristics, medical and obstetric history, and physical examination.

Participants with signs and/or symptoms suggestive of active TB, and all HIV-positive women (irrespective of clinical presentation), were asked to submit two morning sputum samples for TB liquid culture, GeneXpert MTB/RIF PCR and smear microscopy. In addition, urine was obtained for TB culture in case of pyuria and/or haematuria. Blood samples were obtained for routine antenatal care tests and QFT-Plus at the inclusion visit. For HIV-positive women, CD4+ T cells were measured using flow cytometry (FACS Calibur, Becton Dickinson).

### 2.2 QuantiFERON-TB Gold Plus assay

Venous blood was collected in lithium-heparin tubes and transported to the Adama Regional Laboratory, where antigen stimulations were initiated within 8 hours of venepuncture. One ml of blood was dispensed into each of the four assay tubes (Nil, TB1, TB2, mitogen), followed by mixing by inversion and incubation at 37° C for 16–24 hours (aiming at 18 hours’ incubation time). After incubation, the tubes were centrifuged, and aliquots of the supernatants were stored at -20° C.

IFN-γ ELISA was performed according to the QFT-Plus protocol. ELISA results were converted to international units per millilitre (IU/ml) and interpreted using the software supplied by the manufacturer. All IFN-γ concentrations were nil-corrected. The upper limit of linearity for the assay is 10 IU/ml, consequently, IFN-γ concentrations exceeding 10 IU/ml were designated 10 IU/ml. Results ≥0.35 IU/ml in either of the two antigen tubes were considered to be positive with regard to the primary study objective. Sixteen (1.9%) women had initially indeterminate QFT-Plus test results; on re-testing, results from all these cases were possible to interpret. Seven of these were caused by pipetting errors, 5 were due to low mitogen response and 4 to high nil values.

IFN-γ levels in the two TB antigen tubes were compared, with special focus on discordant results. Concordant and discordant results were categorized based on the cut-off levels for TB1 and TB2 recommended by the manufacturer (0.35 IU/ml). Furthermore, the distribution of values within a borderline zone (defined as 0.20–0.70 IU/ml) were analysed separately. Finally, the lower and upper threshold levels of the borderline range were explored as alternative cut-off values.

### 2.3 Ethical considerations

This study was approved by ethical review committees at the Faculty of Medicine, Lund University, Lund Sweden, and at the Ministry of Science and Technology, Addis Ababa, Ethiopia. Written informed consent was obtained prior to enrolment. QFT-Plus results were not distributed to health providers. Currently, testing and treatment for LTBI is not recommended in antenatal care in Ethiopia. Isoniazid prevention therapy (IPT) has been recommended for HIV-positive subjects in Ethiopia since 2007, after exclusion of active TB [33].

### 2.4 Statistical analysis

For the purpose of this study, the analysis was restricted to factors considered relevant with respect to Mtb antigen reactivity; HIV serostatus, age, parity and gestational age. Agreement between IFN-γ concentrations in TB1 and TB2 was quantified using Kappa-statistics and paired samples Z-test was used to compare the difference between proportions of TB1-/TB2+ and TB1+/TB2- discordance. The association between IFN-γ concentrations in TB1 and TB2 was determined using nonparametric correlation. Bland-Altman plots were constructed to evaluate continuous agreement between TB1 and TB2.

Kruskal-Wallis test was used to compare age, parity and gestational age across QFT-Plus result categories in two sets (concordant negative, TB1+/TB2-, TB1-/TB2+ and concordant positive; and concordant <0.20 IU/ml, concordant 0.20–0.70 IU/ml, and concordant >0.70 IU/ml). In case of significant asymmetry, post-hoc pairwise Mann-Whitney’s U-test was used to localize and assess the association between individual categories. Pairwise Fisher’s test was used to compare proportions of HIV-positive women. To compensate for multiple statistical testing generated by this approach, the Bonferroni method was used, resulting in a level to define statistical significance of p <0.0033 for these analyses.

For comparison of absolute levels of IFN-γ with regard to HIV serostatus, Mann-Whitney’s U-test was used, whereas non-parametric correlation was used for analysis of IFN-γ concentrations in relation to gestational age. For that comparison, women with QFT-plus results ≥0.35 IU/ml were included, whereas all participants with QFT-Plus results ≥0.20 IU/ml were included for comparison of IFN-γ levels with regard to HIV serostatus (due to the lower capacity of IFN-γ secretion in HIV infection). All statistical analyses were performed using IBM SPSS statistics version 24.

## 3. Results

### 3.1 Study participant characteristics

Study participant characteristics are shown in Table 1. Forty-nine women (5.9%) were HIV-positive. Symptoms compatible with active TB were reported by 27 women. Among these, bacteriological sputum results were available in 13 cases (all negative). Spontaneous symptom resolution was reported in almost all (26/27) of these cases. One woman was clinically diagnosed with active pleural TB in connection to the inclusion visit.

**Table 1:** Study participant characteristics categorized by HIV serostatus.

Characteristic	Total n = 829	HIV-negative n = 780	HIV-positive n = 49
**Age (years)**			
<20	67 (8)	66 (9)	1 (2)
20–24	315 (38)	307 (40)	8 (16)
25–29	322 (39)	300 (39)	22 (49)
≥30	125 (15)	107 (14)	18 (37)
Missing data	0	0	0
**Parity**			
0	340 (42)	332 (44)	8 (17)
1	256 (32)	239 (31)	17 (36)
2	128 (16)	119 (16)	9 (19)
>2	83 (10)	70 (9)	13 (28)
Missing data	22/829 (3)	20/780 (3)	2 (4)
**Gestational age**			
Trimester 1	201 (25)	184 (24)	17 (35)
Trimester 2	547 (67)	520 (68)	27 (55)
Trimester 3	69 (8)	64 (8)	5 (10)
Missing data	12/829 (1)	12/780 (2)	0
**HIV serostatus**			
Positive	49 (6)	N/A	N/A
Negative	780 (94)		
**Antiretroviral therapy**			
Yes	N/A	N/A	46 (94)
No			3 (6)
**CD4 count (cells/mm^3^)**			
<200	N/A	N/A	2 (6)
200–399			7 (23)
400–799			16 (52)
≥800			6 (19)
Missing data			18/49 (37)

Distribution of background characteristics among all, HIV-negative and HIV-positive study participants. Valid column percentage is shown in brackets.

### 3.2 Performance of QFT-Plus in pregnant women

#### i) With regard to the recommended cut-off level of 0.35 IU/ml

**Fig 1:**
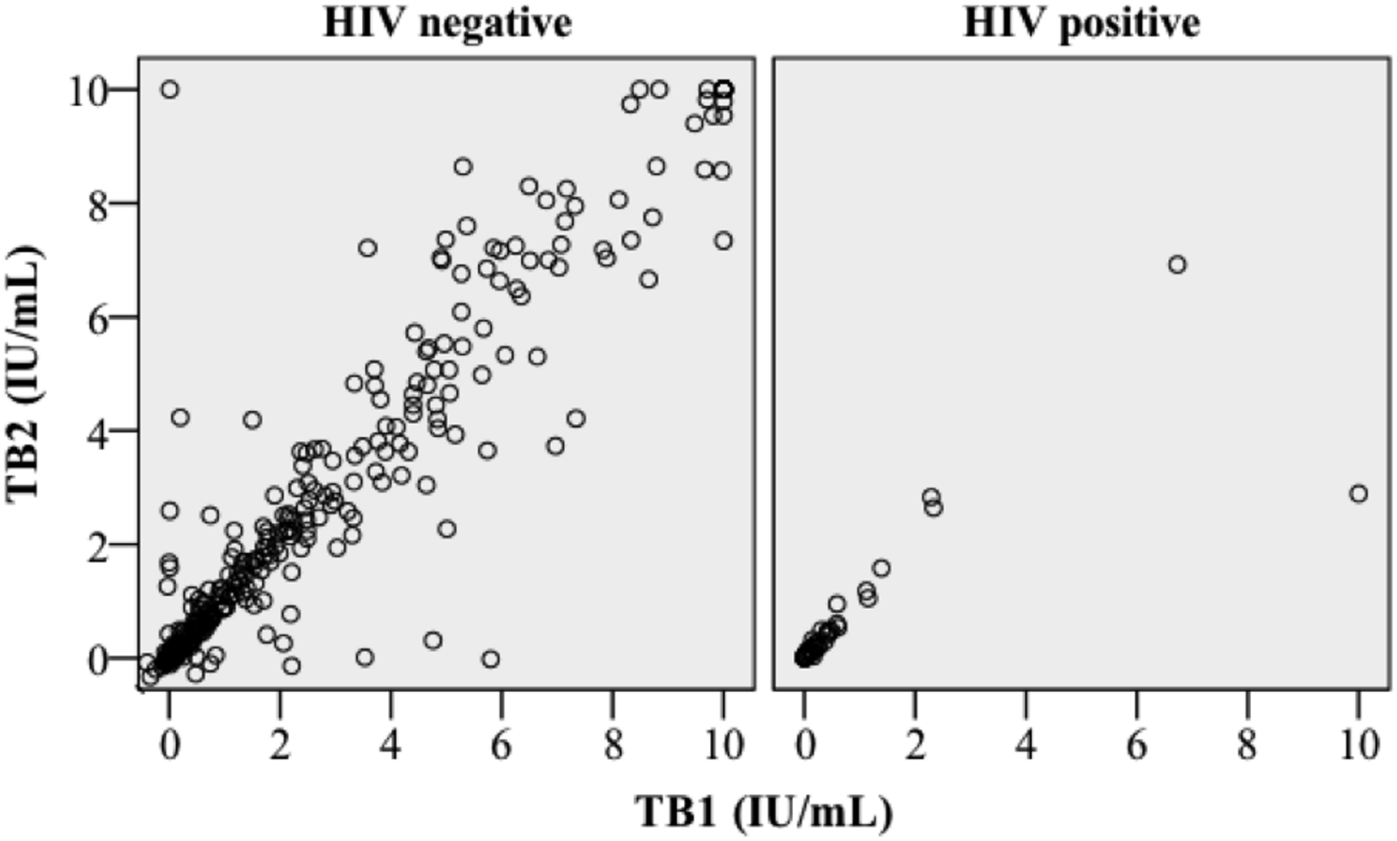
Association of TB1 and TB2 IFN-γ-levels. Scatter-plot of IFN-γ concentrations in TB1 and TB2 divided by HIV serostatus.

Using the cut-off value recommended by the manufacturer, 277/829 (33%) of participants were categorized as QFT-Plus positive (≥0.35 IU/ml in TB1 or TB2). Twenty-nine women (3.5%) had discordant results, of which 16 (1.9%) were TB1+/TB2- and 13 (1.6%) were TB1-/TB2+; 248 (30%) were concordant positive. The agreement between TB1 and TB2 based on this cut-off was high (κ = 0.92, p<0.0001), and the correlation between IFN-γ concentrations in TB1 and TB2 was strong (Spearman’s Rho 0.89; p<0.0001; Fig 1).

Among 780 HIV negative women, 261 (33%) were QFT-Plus positive (IFN-γ ≥ 0.35 IU/ml in either tube), 15 (1.9%) were TB1+/TB2- discordant, 12 (1.5%) were TB1-/TB2+ discordant and 227 (29%) were concordant positive. There was a high agreement (κ = 0.92, p<0.0001), and strong correlation between TB1 and TB2 (Spearman’s Rho 0.88; p<0.0001; Fig 1, S1 Fig). Among 49 HIV-positive women, 16 (33%) were QFT-Plus-positive (IFN-γ ≥0.35 IU/ml in either TB1 or TB2), one (2.0%) was TB1+/TB2- discordant, one (2.0%) was TB1-/TB2+ discordant and 14 (29%) were concordant positive. There was a high agreement between TB1 and TB2 (κ = 0.90, p<0.0001) and a strong correlation between IFN-γ concentrations in TB1 and TB2 (Spearman’s Rho 0.96; p<0.0001; Fig 1 and S1 Fig).

Reflecting the strong correlation between TB1 and TB2, the discordant cases had median IFN-γ concentrations closer to the cut-off value (TB1+/TB2-: TB1 0.49, IQR [0.38–2.17]; TB2 0.24 IQR [-0.01–0.30] IU/ml, TB1-/TB2+: TB1 0.18, IQR [0.01–0.27] IU/ml, TB2 0.49 IQR [0.43–2.14] IU/ml), and consequently had lower median IFN-γ levels than the concordant positive cases (TB1: 2.52 IQR [1.12–5.93] IU/ml; TB2: 2.77 IQR [1.14–6.74 IU/ml]) and higher than the concordant negative cases (TB1: 0.00 IQR [-0.01–0.02] IU/ml; TB2: 0.01 IQR [-0.01–0.03] IU/ml).

The distribution of gestational age, parity and HIV-serostatus was similar across these categories (Table 2), although this comparison was limited by the low number of discordant results. There was a tendency for QFT-Plus positive women to be older than QFT-Plus negative, although this association did not reach statistical significance when adjusted for multiple comparisons (Table 2).

**Table 2:** Characteristics of QFT-Plus-negative, discordant and concordant positive women.

	TB1-/TB2- n = 552	TB1+/TB2- n = 16	TB1-/TB2+ n = 13	TB1+/TB2+ n = 248
**Age (years)**				p = 0.005^1^
<20	50 (75)	0 (0)	0 (0)	17 (25)
20–24	222 (70)	4 (1)	5 (2)	84 (27)
25–29	206 (64)	10 (3)	7 (2)	99 (31)
≥30	74 (59)	2 (2)	1 (1)	48 (38)
Missing data	0	0	0	0
**Parity**				NS^1^
0	238 (70)	4 (1)	6 (2)	92 (27)
1	175 (68)	6 (2)	4 (2)	71 (28)
2	71 (55)	3 (2)	2 (2)	52 (41)
>2	52 (63)	2 (2)	1 (1)	28 (34)
Missing data	16 (72)	1 (5)	0	5 (23)
**Gestational age**				NS^1^
Trimester 1	133 (67)	8 (4)	3 (1)	57 (28)
Trimester 2	367 (67)	5 (1)	10 (2)	165 (30)
Trimester 3	43 (63)	3 (4)	0 (0)	23 (33)
Missing data	9 (75)	0	0	3 (25)
**HIV**		NS^2^	NS^2^^,^^3^	NS^2^^,^^3^^,^^4^
Positive	33 (67)	1 (2)	1 (2)	14 (29)
Negative	519 (66)	15 (2)	12 (2)	234 (30)

^1^Kruskal-Wallis test was used to compare age, parity and gestational age. Proportions of HIV-positive subjects was tested by pairwise Fisher’s test.

^2^Compared with TB1-/TB2-.

^3^Compared with TB1+/TB2-.

^4^Compared to TB1-/TB2+.

(Adjusted level of significance according to the Bonferroni method; p<0.0033).

Non-significant (NS).

Valid row percentage is shown in brackets.

#### ii) With regard to a borderline range (0.20–0.70 IU/ml)

We investigated the distribution of QFT-Plus results within a borderline range compared to results below and above the limits of the borderline range selected for this study (<0.20 and >0.70 IU/ml, respectively). Among 780 HIV negative women, 59 (7.6%) had TB1 borderline results, 59 (7.6%) had TB2 borderline results, and 43 (5.5%) had borderline results for both TB1 and TB2 (Table 3). The respective proportions for HIV-positive women were 14/49 (29%; TB1), 12/49 (24%; TB2) and 11/49 (22%; TB1 and TB2) (Table 4). Compared to HIV-negative women, HIV-positive women were more likely to have borderline results than results <0.20 IU/ml (TB1: OR = 4.3, 95% CI [2.2–8.7], p<0.0001; TB2: OR = 3.6, 95% CI [1.7–7.3], p = 0.0003) and >0.70 IU/ml (TB1: OR = 7.2, 95% CI [2.8–18.5], p = 0.0001; TB2: OR = 5.4, 95% CI [2.2–13.9], p = 0.0004) compared to HIV-negative women. Women with IFN-γ <0.20 IU/ml were slightly younger and there was a non-significant trend of having delivered fewer times than women with borderline or >0.70 IU/ml results; gestational age was similar across categories based on the borderline range (Table 5).

**Table 3:** Distribution of 780 HIV-negative women with respect to the conventional cut off 0.35 IU/ml and a borderline range (0.20–0.70 IU/ml) in TB1 and TB2.

		**TB2 interval (IU/ml)**				
**TB1 interval (IU/ml)**		**<0.20**	**0.20–0.34**	**0.35–0.70**	**>0.70**	**Total**
	**<0.20**	495 (63)	8 (1)	2 (0.3)	5 (0.6)	510 (65)
	**0.20–0.34**	5 (0.6)	11 (1)	4 (0.5)	1 (0.1)	21 (3)
	**0.35–0.70**	2 (0.3)	6 (0.8)	22 (3)	8 (1)	38 (5)
	**>0.70**	5 (0.6)	2 (0.3)	4 (0.5)	200 (26)	211 (27)
	**Total**	507 (65)	27 (3)	32 (4)	214 (27)	780 (100)

Percentage of total (n = 780) shown in brackets.

**Table 4:** Distribution of 49 HIV-positive women with respect to the conventional cut off 0.35 IU/ml and a borderline range (0.20–0.70 IU/ml) in TB1 and TB2.

		**TB2 interval (IU/ml)**				
**TB1 interval (IU/ml)**		**<0.20**	**0.20–0.34**	**0.35–0.70**	**>0.70**	**Total**
	**<0.20**	27 (55)	1 (2)	0 (0)	0 (0)	28 (57)
	**0.20–0.34**	2 (4)	3 (6)	1 (2)	0 (0)	6 (12)
	**0.35–0.70**	0 (0)	1 (2)	6 (12)	1 (2)	8 (16)
	**>0.70**	0 (0)	0 (0)	0 (0)	7 (14)	7 (14)
	**Total**	29 (59)	5 (10)	7 (14)	8 (16)	49 (100)

Percentage of total (n = 49) shown in brackets.

**Table 5:** Characteristics of women with regard to QFT-Plus results in the borderline interval (0.20–0.70 IU/ml).

Characteristic	TB1<0.20/TB2<0.20 n = 522	TB1-BL/TB2-BL n = 54	TB1>0.70/TB2>0.70 n = 207
**Age (years)**		p<0.0001^2^	p<0.0001^1^ p = 0.006^2^, p = 0.018^3^
<20	49 (74)	2 (3)	15 (23)
20–24	215 (73)	9 (3)	72 (24)
25–29	190 (63)	31 (10)	81 (27)
≥30	68 (57)	12 (10)	39 (33)
Missing data	0	0	0
**Parity**			p = 0.004^1^
0	231 (72)	14 (4)	78 (24)
1	163 (67)	21 (9)	57 (24)
2	66 (56)	12 (10)	41 (34)
>2	46 (59)	6 (8)	26 (33)
Missing data	16 (72)	1 (5)	5 (23)
**Gestational age**			NS^1^
Trimester 1	125 (66)	17 (9)	48 (25)
Trimester 2	349 (68)	33 (6)	134 (26)
Trimester 3	39 (60)	4 (6)	22 (34)
Missing data	9 (75)	0	3 (25)
**HIV**		p<0.0001^2^	NS^2^, p<0.0001^3^
Positive	27 (60)	11 (24)	7 (16)
Negative	495 (67)	43 (6)	200 (27)

^1^Kruskal-Wallis test used for age, parity and gestational age. Mann-Whitney’s U-test was used for post-hoc analysis. Proportions of HIV-positive subjects was tested by pairwise Fisher’s test.

^2^Compared with TB1<0.20/TB2<0.20.

^3^Compared with TB1BL/TB2BL.

(Adjusted level of significance according to the Bonferroni method p<0.0033).

Non-significant (NS), borderline (BL [IFN-γ 0.20–0.70 IU/ml]).

Valid row percentage is shown in brackets.

Due to the lack of gold standard for LTBI, the optimal cut-off cannot be precisely determined and may be influenced by immune status and likelihood of TB exposure. Therefore, we wanted to explore the borders of the borderline interval as potential alternative cut-off values depending on the setting. Using the upper limit of the borderline zone for defining a positive QFT-Plus result (>0.70 IU/ml), 225/780 HIV negative (29%) and 8/49 HIV positive (16%) women were classified as positive in either TB1 or TB2 (Tables 3 and 4). Defining positivity using the lower borderline limit (≥0.20 IU/ml), 285/780 HIV-negative (37%) and 22/49 HIV-positive (45%) women were classified as positive in either TB1 or TB2 (Tables 3 and 4).

### 3.3 Absolute IFN-γ responses with regard to HIV serostatus

**Fig 2:**
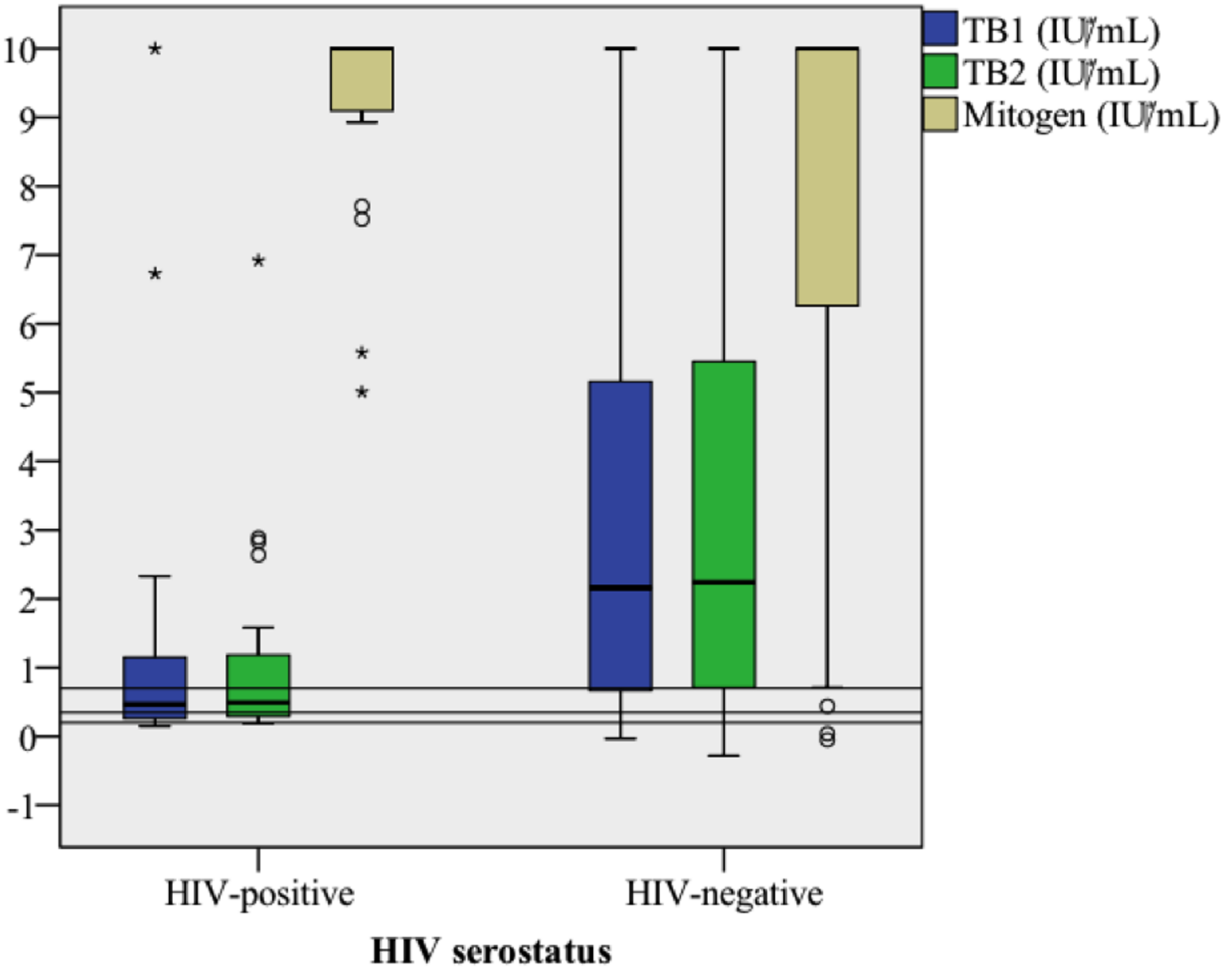
Box-plot of IFN-γ release from TB1 and TB2 antigen formulations- and mitogen stimulation compared by HIV-serostatus. HIV-positive (n = 22) and HIV-negative (n = 285) study subjects with IFN-γ≥0.20 IU/ml in at least one TB antigen formulation were included. Horizontal lines represent borders of the borderline zone (0.20 and 0.70 IU/ml) and the conventional cut-off (0.35 IU/ml).

We hypothesized that HIV infection would impact absolute levels of IFN-γ after Mtb stimulation in women sensitized to Mtb, and therefore compared these levels between HIV-positive and -negative pregnant women. Due to their reduced capacity to produce IFN-γ, all women with IFN-γ response ≥0.20 IU/ ml (the lower threshold level in the borderline range) in at least one tube were included in this analysis. Among 22 HIV positive and 285 HIV negative women, both median TB1- and TB2-stimulated IFN-γ were significantly lower in HIV-positive than HIV-negative individuals (TB1: 0.47 vs. 2.16 IU/ml; p<0.01, TB2: 0.49 vs. 2.24 IU/ml; p<0.001: Fig 2).

### 3.4 Mitogen and TB-specific IFN-γ responses with regard to gestational age

**Fig 3:**
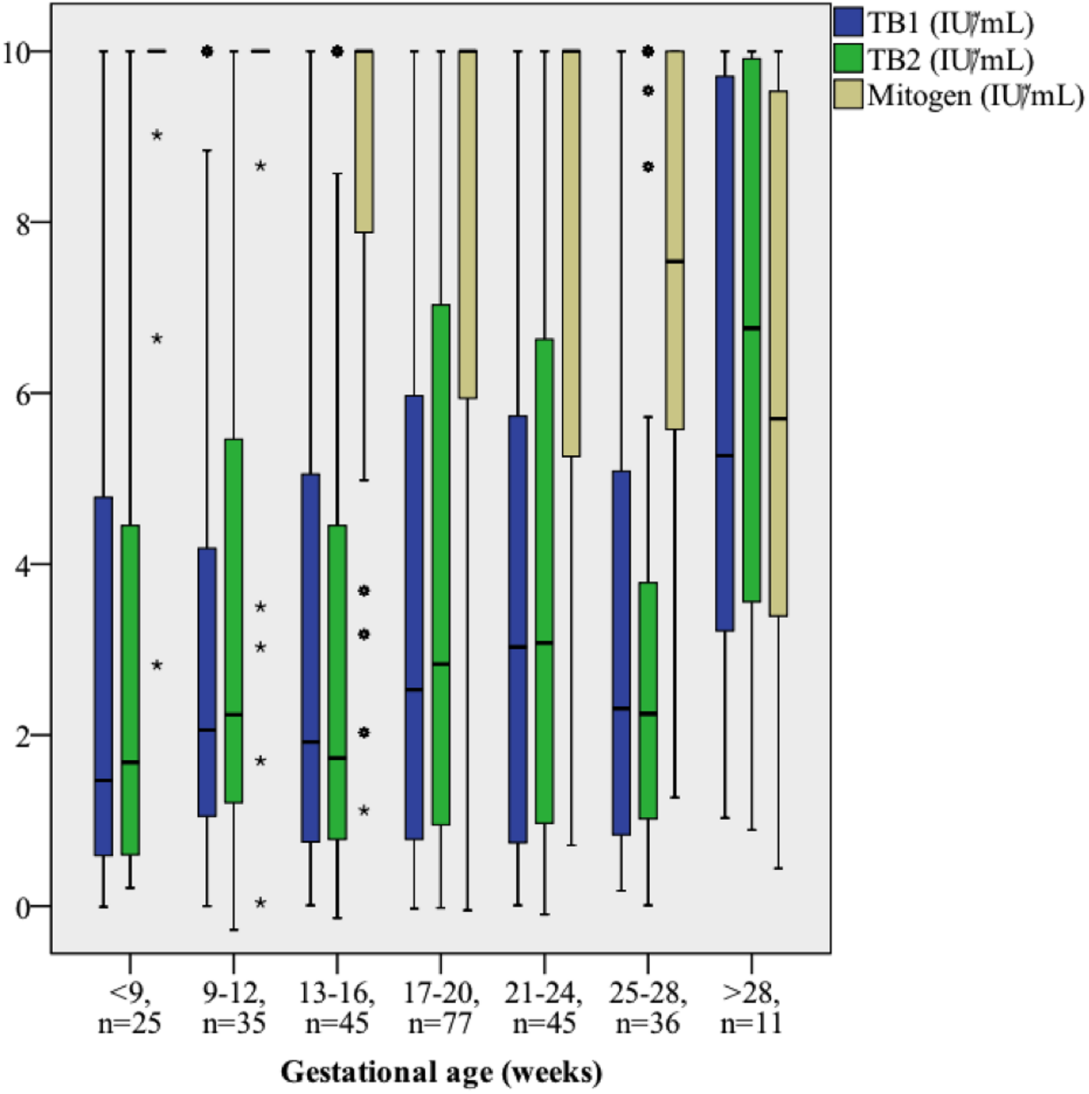
Mitogen and antigen-stimulated IFN-γ response stratified by gestational age. Box-plot of IFN-γ concentrations in QFT-Plus positive women. Concentrations exceeding 10 IU/ml were without the linear range and were designated to 10 IU/ml.

We hypothesized that IFN-γ secretion might decrease in relation to gestational age due to increasing pregnancy-related immune suppression [3,4,17–21]. For this purpose, we investigated Mtb response at different stages of pregnancy by comparing IFN-γ levels in QFT-Plus-positive women with LTBI (cut-off level 0.35 IU/ml) sampled at different gestational ages. Mitogen-stimulated IFN-γ concentrations were lower among women sampled later compared to those sampled early in pregnancy (Spearman’s Rho -0.32, p<0.0001; Fig 3). In contrast, TB1 and TB2 antigen stimulation elicited slightly stronger IFN-γ response in women who were tested later in pregnancy (Spearman’s rho 0.13 and 0.11; p = 0.031 and p = 0.061, respectively). Although these trends were also observed for HIV-positive and—negative women separately, they did not reach independent statistical significance. The proportion of positive QFT-Plus test results was similar regardless of trimester at sampling (Table 2).

## 4. Discussion

In this study, QFT-Plus indicated TB infection in 33% of pregnant Ethiopian women. The addition of short peptide antigens that stimulate both CD4+ and CD8+ T cells (TB2) led to detection of LTBI in 13/829 (1.6%) cases. Another subset of women (16/829; 1.9%) showed positive reaction only after TB1 stimulation. However, the overall agreement between QFT-Plus results for TB1 and TB2 antigens was high, with similar median IFN-γ levels with the two antigen formulations, also among HIV-positive women. The proportions of QFT-Plus positive subjects (using 0.35 IU/ml as cut-off level) were similar among HIV–positive and HIV–negative individuals. IFN-γ levels were lower in subjects with discordant results compared to women with concordant positive results. Furthermore, HIV-positive subjects had lower median IFN-γ responses, and a higher proportion of HIV-positive women had QFT-Plus results within a borderline range for both TB1 and TB2. To our knowledge, the distribution of QFT borderline results has not previously been assessed in pregnant women. For this study, we chose to use a borderline range of 0.20–0.70 IU/ml, which recently was evaluated for QFT-GIT with respect to TST concordance and incidence of active TB [31].

Importantly, the interpretation of borderline results depends both on the likelihood of TB exposure and the presence of immunosuppression, which is also associated with an increased risk of LTBI reactivation. In our study population, residing in a TB-endemic region, a substantial proportion had QFT-Plus borderline results (73/829 [8.8%] in TB1 and 71/829 [8.6%] in TB2). Interestingly, HIV-positive women were more likely to have Mtb stimulated IFN-γ levels within the borderline range compared to HIV-negative women, and these individuals also had lower absolute levels of TB antigen stimulated IFN-γ release. The lower median IFN-γ levels found in HIV-positive subjects could reflect lower absolute numbers of CD4+ T-cells [34], but also the reduced capacity for IFN-γ secretion in HIV infection [11]. This phenomenon may also explain higher rates of indeterminate QFT results observed in other studies [35], and lower rates of positive QFT-GIT results observed in HIV-positive subjects despite higher rate of TB exposure compared to HIV-negative controls, as reported by Lin et al [36]. For these reasons, it is possible that results in the lower range of the borderline zone may represent true TB infection in HIV-positive subjects, although this hypothesis could not be confirmed with the design of this study, and due to the lack of a gold standard method for determination of LTBI. Our findings are in agreement with results on repeated QFT-GIT testing in HIV-positive subjects after TST administration, suggesting a cut-off level of 0.15 IU/ml to define positive QFT results in such individuals [32]. Further studies are needed to investigate whether a lower threshold level to define positive QFT-Plus results in immunosuppressed individuals should be introduced to improve sensitivity.

Furthermore, the immune modulation that occurs during the course of pregnancy might influence the performance of diagnostic tests for LTBI. Decreasing IFN-γ secretion was observed both for Mtb and mitogen stimulation during the course of pregnancy in Indian HIV-positive women [15], and among HIV-positive Kenyan women, both mitogen and Mtb stimulated IFN-γ response increased post-partum compared to those observed during pregnancy [13]. In line with this, QFT-GIT conversions in HIV-negative women tested longitudinally during pregnancy and post-partum suggest that TB antigen-elicited IFN-γ response may be attenuated during pregnancy [14]. Apart from HIV infection, helminth infection has also been suggested to affect QFT-GIT response during pregnancy, and has been associated with indeterminate results in pregnant Ethiopian women [37]. In our study, we found significantly lower levels of mitogen induced IFN-γ, but also slightly higher levels of Mtb antigen stimulated IFN-γ, in women tested at later stages of pregnancy. This phenomenon could be explained by increased Mtb stimulation during the course of pregnancy, which in turn might be caused by asymptomatic relative reactivation of LTBI. However, it is impossible to exclude that this finding is a result of confounding, and since we did not obtain serial samples during pregnancy from these participants, these results have to be interpreted cautiously. Further studies are required to elucidate these mechanisms and their potential association with incident TB.

To our knowledge, this is the first evaluation of the performance of QFT-Plus for the diagnosis of LTBI in pregnancy. This study was performed in a representative public health care setting in a region with high TB prevalence, and 5.9% of participants were HIV-positive. Apart from a study on patients with suspected active TB in Zambia [38], no other evaluations of QFT-Plus have hitherto been conducted in TB-endemic regions. Furthermore, women with clinical manifestations suggestive of active TB underwent systematic bacteriological investigations with prospective follow-up.

Our study has several limitations. We did not include comparison with TST and QFT-GIT; the latter comparison could have shown the effect of omission of the TB7.7 antigen. However, previous comparisons found high agreement and similar or higher rates of test positivity for QFT-Plus compared to QFT-GIT with respect to active tuberculosis [39,40], and LTBI [39,41–43], suggesting that the sensitivity of QFT-Plus is at least similar to that of QFT-GIT. Since participants were only tested once, longitudinal comparisons for individual cases were not possible. Such follow-up testing might be particularly relevant for subjects with borderline results at initial testing [29]. Despite a relatively high number of HIV-positive women in the study population, the statistical power was insufficient to determine the performance of QFT-Plus in this subset of pregnant women. Furthermore, we did not include a control population of non-pregnant women. Although our data suggest the use of a lower cut-off level to define positive QFT-Plus results in immunosuppressed individuals, we have not explored whether 0.20 IU/ml is optimal for this purpose; it is possible that an even lower threshold level would be more sensitive [32]. In future studies of TB infection in relation to pregnancy these limitations should be addressed. Finally, data on certain variables were missing from a proportion of participants; however, this proportion was rather small, and we consider it unlikely that this would have affected our overall results.

## Conclusion

We found high agreement between QFT-Plus results elicited by TB1 and TB2 antigen formulations. No particular characteristics distinguished subjects with TB1/TB2-discordant results. IFN-γ responses among HIV-positive women were similar for the two antigen formulations, but were significantly lower than those in HIV-negative persons, suggesting that a lower cut-off might be considered to define positive QFT-Plus results for HIV-positive pregnant women.

## Supporting information

S1 Fig Bland-Altman plot divided by HIV serostatus. TB2 minus TB1 plotted against the mean of TB1 and TB2.
(PDF)

S1 SPSS File SPSS file containing all data on which this manuscript is based. (SAV)
