# Supplementary figures and images for: Performance of QuantiFERON-TB Gold Plus for detection of latent tuberculosis infection in pregnant women living in a tuberculosis- and HIV-endemic setting
Source: PLoS One. 2018 Apr 4;13(4):e0193589. doi: 10.1371/journal.pone.0193589 (PMC5884484; doi:10.1371/journal.pone.0193589)

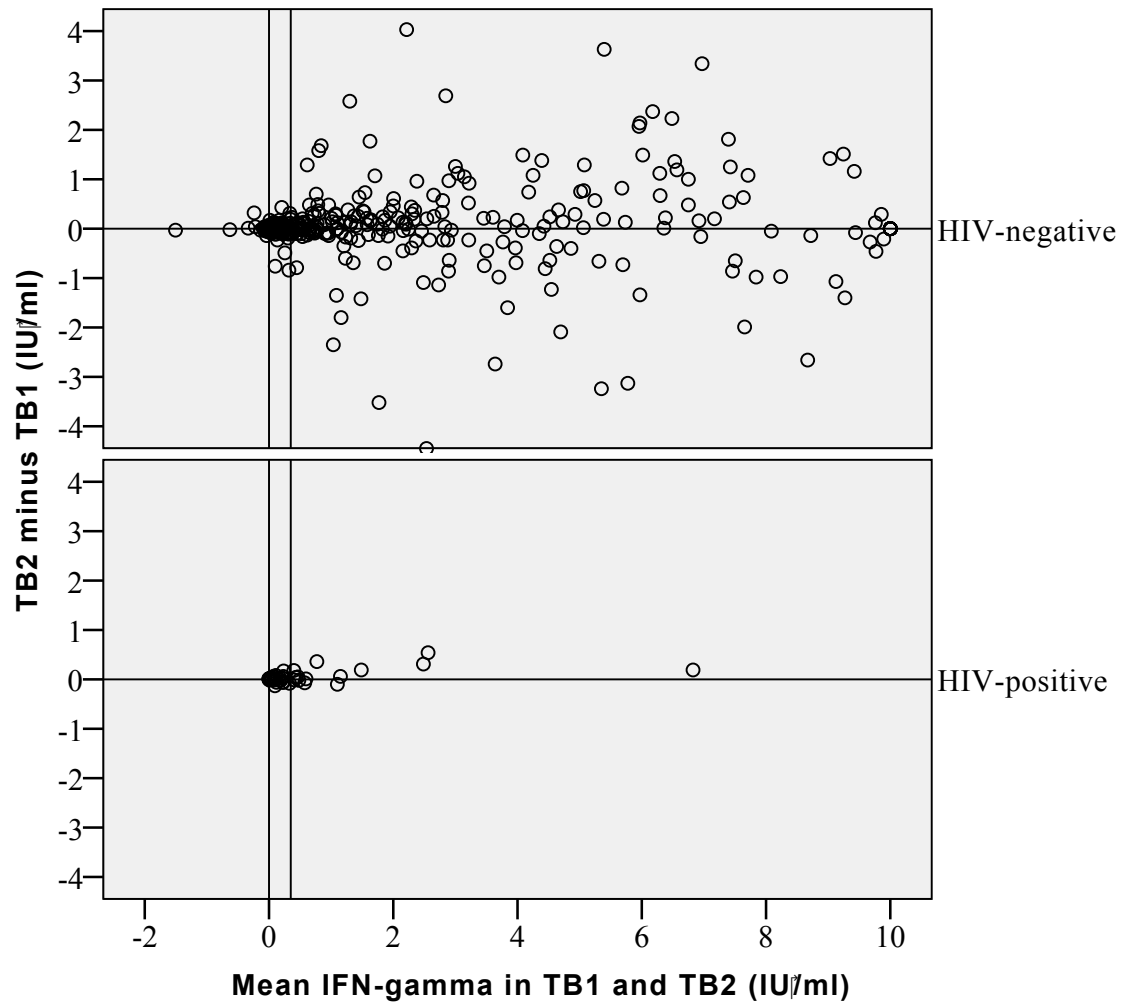

Supplement: S1 Fig — TB2 minus TB1 plotted against the mean of TB1 and TB2. (PDF) [file pone.0193589.s001.pdf]
